# Effects of a single dose of L-histidine on mental fatigue and vigor in participants with high fatigue levels: a randomized controlled trial

**DOI:** 10.1038/s41598-026-48060-x

**Published:** 2026-04-15

**Authors:** Masatsugu Shimomasuda, Haruna Fujiyoshi, Yu Kajiyama, Mayuka Kanda, Kenta Kajiwara, Kenji Nagao, Akira Abe, Shigekazu Kurihara, Hiroyuki Kato, Hidehiro Nakamura

**Affiliations:** 1 Research Institute for Bioscience Products & Fine Chemicals, Ajinomoto Co., Inc., Kawasaki-shi, Japan; 2 Business Strategy and Development Department, Ajinomoto Co., Inc., Chuo-ku, Japan; 3 Amino Acids Department, Ajinomoto Co., Inc., Chuo-ku, Japan; 4 Sports & Health Nutrition Department, Ajinomoto Co., Inc., Chuo-ku, Japan

**Keywords:** L-histidine, Single dose, Mental fatigue, High fatigue levels, POMS2, Negative mood states, Diseases, Health care, Medical research, Neuroscience, Psychology, Psychology

## Abstract

Mental fatigue is a psychobiological state caused by prolonged periods of demanding cognitive activity that affects many aspects of daily life. Two weeks of L-histidine daily ingestion in humans significantly improves mood states compared with a placebo; however, the effects of a single dose are unknown. We aimed to assess the effects of a single dose of L-histidine on mood states, including fatigue, in healthy men and women who regularly experienced fatigue and sleep deprivation. Participants were randomly assigned to the L-histidine or placebo group. They were provided a workload using the Uchida–Kraepelin Performance test, their mood states before and after the workload were assessed, and performance on mental tasks was measured. In the all participants analysis set, the results did not show superiority of the L-histidine group over the placebo group for all endpoints. However, in the subgroup with high fatigue levels, the L-histidine group showed significant decreases compared with the placebo group in the changes in fatigue-inertia (FI), confusion-bewilderment (CB), depression-dejection (DD), and total mood disturbance (TMD) T-scores and a significant increase in the change in vigor-activity (VA) T-score (*p* = 0.022, 0.049, 0.039, 0.036, and 0.023, respectively). Although a single ingestion of L-histidine showed no significant improvements in any of the endpoints compared with the placebo in the prespecified all-participants analysis set, the exploratory findings suggest that it may alleviate negative mood states, including fatigue, confusion, and depression, and may also improve vigor after a workload in humans with high fatigue levels. Clinical Trial Registry number: UMIN000055719.

**Supplementary Information:**

The online version contains supplementary material available at 10.1038/s41598-026-48060-x.

## Introduction

Mental fatigue has become a social problem in recent years. Individuals from various backgrounds, including business professionals, students, and homemakers, constantly experience mental strain in their daily lives, and many experience mental fatigue and lack of vigor^1^. Mental fatigue is a psychobiological condition caused by prolonged periods of demanding cognitive activity that affects many aspects of daily life. It manifests itself as subjective symptoms (e.g., increased mental fatigue, lack of vigor, decreased motivation, and decreased attention), and mental fatigue can lead to poor performance in cognitive tasks and induce sleep disorders^2–6^.

Histamine is involved in various brain functions. In mammals, the central histaminergic neurons are located in the posterior hypothalamus and project their axons onto the central nervous system^7^. Histamine in the brain is produced by the tuberomammillary nucleus in the posterior hypothalamus or mast cells in different parts of the brain and acts as a neurotransmitter via four types of histamine receptors (H1R, H2R, H3R, and H4R)^8^. Each of these receptors has a distinct function: H1R specifically regulates behavior, nutritional status and various physiological functions, including arousal, appetite, cognition, and emotions; the action of H2R is typically excitatory; H2R agonists enhance synaptic transmission in the hippocampus, a brain structure important for long-term memory and spatial navigation, and increase the firing rate of many types of neurons; and H3R functions as an autoreceptor, regulating the release of several neurotransmitters, including histamine, serotonin, gamma-aminobutyric acid, and glutamate^7–12^.

L-histidine is a histamine precursor; therefore, dietary ingestion of L-histidine is thought to play an important role as a source of histamine in the brain. In mammals, orally ingested L-histidine is absorbed into the bloodstream from the small intestine and crosses the blood–brain barrier^13–15^. These findings suggest that L-histidine ingestion increases brain histamine levels and potentially reduces mental fatigue. Certainly, animal studies have supported this; sleep deprivation by water-floor stress in male mice has been reported to increase histamine consumption, leading to decreased histamine levels in the brain and impaired working memory in the Y-maze test^16^. The results showed that a single oral ingestion of L-histidine increased extracellular histamine concentrations around the prefrontal cortex (PFC) and basal forebrain, resulting in an increase in the number of c-Fos-positive cells around these areas, followed by attenuation of memory impairment. Increased c-Fos-positive cells are involved in neural activity, neural circuit reorganization, and cognitive functions, such as learning and memory. Furthermore, this beneficial effect of L-histidine on memory has been abolished by intracerebroventricular injection of α-fluoromethylhistidine, a histidine decarboxylase inhibitor. These results suggest that a single oral dose of L-histidine replenishes histamine in the brain and reverses sleep deprivation-induced memory impairment via histaminergic activation.

The effects of L-histidine daily ingestion on mental fatigue have also been confirmed in humans. In a prior crossover study, 20 men aged 45–65 years who regularly experienced mental fatigue and sleep deprivation were administered L-histidine (1.65 g once daily for 2 weeks) and showed significant improvements compared with the placebo group in subjective assessments of mental fatigue, clear thinking, attention, and reaction time in a visual learning task^17^. Dried bonito broth (DBB) is a popular food ingredient traditionally used in various Japanese dishes, and its ingestion improves mental fatigue, mental task performance, and mood in daily life^18–22^. DBB contains abundant quantities of histidine, which makes up approximately 10% of its dry matter^23^, suggesting that some of the effects of DBB may be associated with L-histidine.

Previous studies have demonstrated the effects of L-histidine daily ingestion in humans. However, we speculated that an acute effect is also expected. The effect of L-histidine on mental fatigue is thought to be due to increased brain histamine^17^. In addition, oral amino acids are absorbed into the blood from the small intestine, and L-histidine crosses the blood–brain barrier in mammals^12–15^. After histidine ingestion, blood histidine reaches its peak concentration in humans and rats approximately 30 to 60 min later^16,24,25^. Furthermore, in rats, histidine in the cerebrospinal fluid and histamine in the prefrontal cortex rise significantly after approximately 60 min, followed by a gradual increase, and a single oral ingestion of L-histidine improved sleep deprivation-induced memory impairment^16^. However, there are no research reports on the effects of a single dose of L-histidine in humans. Thus, we hypothesized that under similar conditions as in mice^16^, i.e., in people experiencing mental fatigue and sleep deprivation, a single dose of L-histidine may have an acute effect on temporarily the negative mood states, such as reducing fatigue and loss of vigor, caused by workload. Therefore, we aimed to assess the effects of a single dose of L-histidine on mood states, including mental fatigue and vigor, in healthy men and women who regularly experienced mental fatigue and sleep deprivation.

## Materials and methods

### Participants

The participants included healthy men and women aged 30–60 years who regularly experienced mental fatigue and sleep deprivation, which were subjective symptoms reported by the participants, and this study did not include those with illnesses. We excluded people who were currently using any medication or herbal medicine; those who regularly use supplements or health foods for fatigue recovery or sleep improvement and cannot stop taking them during this study period; those who regularly consume oily fish more than twice a week, those with a significant chronic disease or those undergoing treatment; those with a body mass index (BMI) of ≥ 30 kg/m^2^ at the time of this screening test; those who are smokers or have quit smoking < 1 year previously; those with an irregular lifestyle, such as shift work and night shift work; those who are participating or intending to participate in a study involving the ingestion of other foods or drugs or the application of cosmetics or drugs; those who have experienced a loss of a family member or pet within the past 3 months; those with a current or past history of food allergies; those with severe menopausal symptoms or symptoms of premenstrual syndrome or premenstrual dysphoric disorder; those who are good at or consider themselves to be fond of calculations or mental arithmetic; those who have previously experienced vasovagal reflex, those who are currently pregnant or breastfeeding; those who wish to become pregnant during the study period; and others deemed unsuitable as study participants by the principal investigator. Among the eligible participants based on the screening test, those with high fatigue levels (Profile of Mood States 2nd Edition-Short [POMS2-S] fatigue-inertia [FI] T-score), high levels of sleep deprivation (Japanese version of the Athens Insomnia Scale [AIS-J]), and low nutritional laboratory test values (hemoglobin, hematocrit, total protein, and albumin levels) were prioritized for inclusion in this study. This study was registered with the University Hospital Medical Information Network (UMIN000055719). The current study was approved by the Human Subjects Research Ethics Committees of Ajinomoto Co., Inc. (ethical approval code: 2024–011) and Suda Clinic Institutional Review Board, Hakusuikai Medical Corporation (ethical approval code: 2024–021). After the purpose, procedures, and schedule of this study were explained, written informed consent was obtained from all the participants in accordance with the Declaration of Helsinki. There were no changes in the protocol after trial commencement. A total of 100 participants were enrolled in this study and randomly assigned by the randomizer to two groups, L-histidine and placebo, in a 1:1 ratio. Covariate-adaptive randomization was performed. Allocation adjustment factors included age, sex, POMS2-S FI T-score, POMS2-S vigor-activity (VA) T-score, and sleep score (AIS-J) at the time of the screening test. The allocation information for each participant was not disclosed to all researchers until the allocation list was opened, which was sealed by the person responsible for allocation and kept sealed until unblinding. This study was conducted using a system that ensured blinding of the participants and all researchers. Furthermore, data management personnel and statistical analysts were blinded to the test samples ingested by the participants to eliminate bias. As there were no existing reports evaluating the effect of a single dose of L-histidine on POMS2-S scores, a sample size design was used based on a previous study^26^ that evaluated the effect of food ingredient ingestion on POMS (pre-revision of POMS2), resulting in a calculated sample size of 37 participants per group (two-sided significance level, 5%; statistical power, 80%). Furthermore, considering the possibility of dropouts and exclusions from the analysis, the number of participants was set to 50 per group (100 participants in total).

### Test samples

The test samples were prepared as follows: A 1.65-g quantity of L-histidine (Ajinomoto Co., Inc., Tokyo, Japan) was compressed into six hard capsules. To prepare the placebo, the cellulose was compressed into six hard capsules. All the test samples were packaged in identical aluminum pouches. The L-histidine and placebo samples were indistinguishable in appearance. After arriving at the research institution, the participants ingested a single dose of 6 L-histidine or placebo capsules with a glass of water (approximately 180 mL) at a specified time.

### Study protocol

**Fig. 1:**
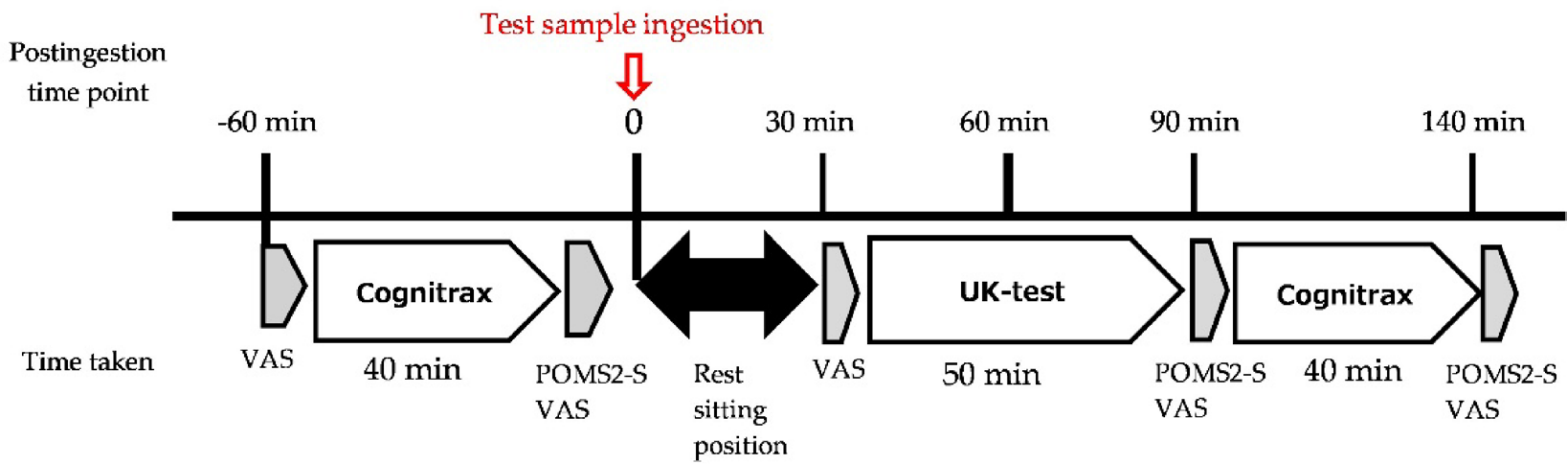
Overview of main exam. The participants ingested the single dose of the test sample (L-histidine or placebo) and were then provided a simple calculation task using the UK-test. Mood states, such as mental fatigue and lack of vigor, and performance on mental tasks were assessed using the POMS2-S, VAS, and Cognitrax. The primary endpoint, POMS2-S, was measured three times: immediately before, 90 min after, and 140 min after test sample ingestion. Baseline values were measured immediately before the ingestion of the test sample, and the primary postingestion assessment was performed 90 min after ingestion.

This study was conducted as a randomized, double-blind, placebo-controlled study. Participants who consented to participate in this study visited the research institution, Higashi-Koganei Sakura Clinic, Hiro-o-kai Medical Corporation, and underwent a screening test that included a medical interview, lifestyle questionnaire completion, anthropometric measurements (height and weight), blood and urine tests, and completion of questionnaires, such as the AIS-J and POMS2-S. Eligible participants were enrolled in the main study, which involved consumption of the test sample (Main Exam), several weeks after the screening test. According to the outline of this study (Fig. 1), the participants ingested a single dose of the test sample (L-histidine or placebo) and completed a simple calculation task using the Uchida–Kraepelin Performance test (UK-test). The POMS2-S, visual analog scale (VAS), and Cognitrax were used to assess mood states, including mental fatigue, lack of vigor, and performance on cognitive tasks. All participants were interviewed during the screening tests and the Main Exam, and subjective and objective findings were confirmed.

### Study endpoints

The primary endpoint of this study was the POMS2-S. The primary endpoint was divided into primary and secondary indices. Primary indices included FI and VA, whereas secondary indices included anger-hostility (AH), confusion-bewilderment (CB), tension-anxiety (TA), depression-dejection (DD), friendliness (F), and total mood disturbance (TMD). The secondary endpoints included VAS score, cognitive function performance using Cognitrax (normalized score), number of answers, number of correct answers, and percentage of correct answers calculated from the UK-test. Safety endpoints included the incidence of adverse events and side effects. The full analysis set (FAS) was used for efficacy evaluation, excluding participants who did not take the test sample and had no post allocation data. The safety analysis set (SAS), which consisted of all participants who took the test sample and were not deemed necessary for exclusion by the principal investigator, was used for safety evaluation.

### Assessments of mood states

Mood states, such as mental fatigue and vigor, were assessed using the POMS2-S (Kaneko Shobo Publishing, Tokyo, Japan) and VAS. The POMS2-S presents 35 questions, and participants respond to each question on a 5-point scale to indicate how well each adjective describes their current mood (0, not at all; 1, a little; 2, moderately; 3, quite a bit; and 4, extremely frequently). The questions covered seven mood factors: FI, AH, CB, DD, TA, VA, and F. In this study, T-scores were used to assess the participants’ mood states. The T-scores were calculated using a T-score conversion table (Kaneko Shobo, Tokyo, Japan). The average T-score for the Japanese participants is 50. Higher T-scores for FI, AH, CB, DD, and TA and lower T-scores for VA and F indicate a more negative mood state. TMD was calculated by adding the T-scores for FI, AH, CB, DD, and TA and subtracting the T-score for VA. The POMS2-S was administered three times: immediately before, 90 min after, and 140 min after the test sample ingestion. The baseline value was measured immediately before ingestion, and the primary postingestion assessment was performed 90 min after ingestion.

Nine questions on the VAS were used to assess the participants’ subjective mood state (positive feelings, clear thinking, motivation, concentration, persistence, energy, progress, relaxation, and tiredness). The VAS consisted of a 10-cm line drawn from one extreme to the other (“best state” [0 cm] to “worst state” [10 cm]), and the participants marked the point on the line that represented their mood state at that time. The VAS was administered five times: 60 min before, immediately before, 30 min after, 90 min after, and 140 min after ingestion. The baseline value was the value immediately before the ingestion of the test sample, and the time point of primary postingestion assessment was 90 min after ingestion.

### Cognitive assessments

Cognitive status was assessed using Cognitrax (Health Solutions, Tokyo, Japan), a computer-based, test based on existing cognitive function tests. The CNS Vital Signs test, on which Cognitrax is based, is a widely recognized and clinically used neuropsychological test. Numerous studies have been published, including the evaluation of test interventions and discriminant validity of mild cognitive impairment (MCI), dementia, and depression compared with healthy controls^27–31^. Three battery tests were available, depending on the purpose of the test. In this study, a basic test (30–40 min) was used to assess general cognitive function. The participants performed seven tasks in the following order: verbal memory, visual memory, finger tapping, symbol digit coding, stroop test, shifting attention, and continuous performance. Based on the test results, the following 12 indices were calculated using a prescribed scoring method and used as endpoints: (a) Neurocognitive Index (NCI), overall score of cognitive function; (b) composite memory, ability to remember (words and figures); (c) verbal memory, ability to remember (words); (d) visual memory, ability to remember (figures); (e) psychomotor speed, ability to quickly process visual information; (f) reaction time, ability to respond quickly to instructions; (g) complex attention, ability to maintain attention and respond accurately; (h) cognitive flexibility, ability to respond to changes in instructions; (i) processing speed, ability to quickly process information; (j) executive function, ability to understand rules and concepts and make decisions; (k) simple attention, ability to pay attention for long periods of time; and (l) motor speed, ability to quickly repeat small movements. These 12 indices were normalized using a normal distribution based on actual measurements, with a mean of 100 and a standard deviation of 15. The “normalized score” was used as the index (values converted for comparison with people of the same age). Cognitrax was performed before (after VAS assessment 60 min before ingestion: baseline) and after (after POMS2-S and VAS assessment 90 min after ingestion) ingestion of the test sample.

### Amount and accuracy of simple tasks

The UK-test (Nisseiken, Inc., Tokyo, Japan) was designed to impose a workload, and the workload and accuracy of the simple tasks were evaluated based on the number of answers, number of correct answers, and percentage of correct answers (i.e., the ratio of correct to total answers). The participants performed simple single-digit addition problems, switching to a new line every minute. With a break in between, the task was performed for 15 min in the first half and 15 min in the second half, for a total of 30 min. The UK-test began after VAS assessment 30 min after the ingestion of the test sample. The results from the first and second halves were compared.

### Statistical analyses

Figures 3, 4, and Fig. S3 are presented as point estimates (Least squares mean differences in each indices with the L-histidine group, compared with the placebo group) and their confidence intervals; all other values are expressed as the means (standard deviations). As specified in the statistical analysis plan, an analysis of covariance was performed on the change from baseline for the POMS2-S (T-score), VAS, and Cognitrax scores, with the test sample group as a fixed effect and the baseline value as a covariate. For the UK-test, the actual measured values of the number of responses, number of correct responses, and correct response rate (first-half total score, second-half total score) were compared between test sample groups using a two-sample t-test. To adjust for multiplicity, a fixed sequence procedure was used for the primary indices, testing the POMS2-S FI and VA, in that order. The time point of primary postingestion assessment was pre-specified as 90 min after ingestion. The statistical analysis plan stipulated that subgroup analyses would be performed if necessary. Based on the results of previous study^16,17^, it was thought that participants with high fatigue levels would be more likely to benefit from L-histidine,thus, an additional analysis was performed on a subgroup limited to participants with a POMS2-S FI T-score ≥ 60 at baseline (defined as “high” fatigue level in the POMS2-S manual). In this subgroup analysis, an analysis of covariance was performed on the change from baseline values, with the test food group as the fixed effect and the baseline value as the covariate. This subgroup analysis was conducted in an exploratory manner after unblinding, and no adjustment for multiplicity was applied. The study was blinded until statistical analysis, and no interim analyses were performed. The analysis was performed without missing data imputation, using data observed at the time of analysis. Statistical analyses were performed using R version 4.2.2. Statistical significance was set at *p* < 0.05.

## Results

### Participants

**Fig. 2:**
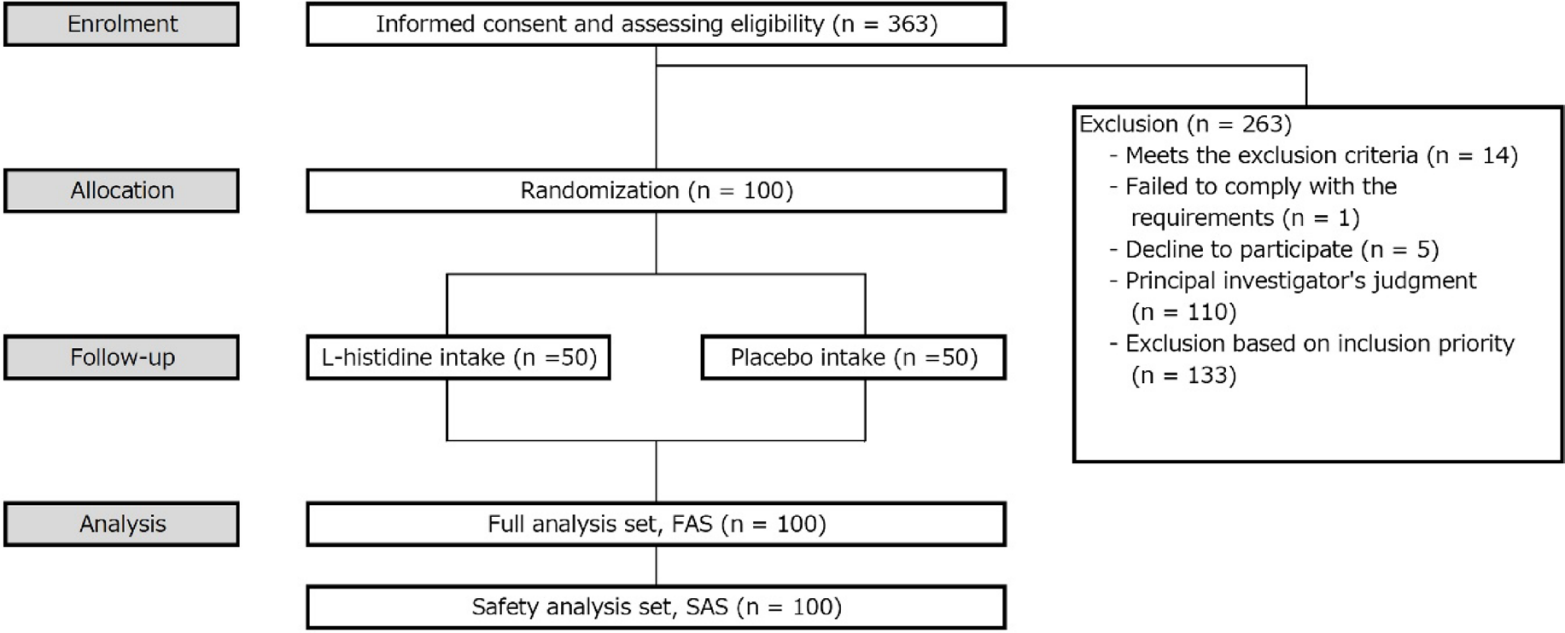
Consolidated standards of reporting trials flow diagram.

The participants underwent screening tests between October 14 and 20, 2024, and subsequently participated in the Main Exam, between November 18 and 22, 2024. Of the applicants for study participation, 363 met the recruitment criteria, provided consent, and underwent screening tests. From the screening test, 100 participants who met the inclusion criteria and did not violate the exclusion criteria were selected and assigned to each group. All 100 participants completed this study, and none discontinued it. The FAS and SAS comprised 100 participants (50 in each group), representing the entire enrolled population. The flow of the study participants from consent to analysis is illustrated in Fig. 2. Prior to unblinding, a case review meeting was held, and some data were excluded from the analysis because of missing data or events that may have affected the data (see the legends of each table for details). Baseline data for the participants’ age, BMI, sleep duration, POMS2-S FI and VA T-scores, nutritional laboratory test values, and nine VAS items are shown in Table 1. The mean T-scores for FI and VA on the POMS2-S were “average” in the category classification indicated in the POMS2 manual in both groups, and all nutritional laboratory test values were within the reference range.

**Table 1:** Baseline data for the study participants.

	Time-point	L-histidine		Placebo	
		n	Mean (SD)	n	Mean (SD)
Parameter					
Number	Main Exam		50		50
Age (years old)	Screening	50	46.7 (8.0)	50	45.9 (7.4)
BMI (kg/m^2^)	Main Exam	50	21.6 (2.2)	50	21.5 (2.4)
Sleep time (min)	The night before Main Exam	50	401.6 (56.6)	50	391.8 (49.3)
POMS2-S T-Score					
Fatigue-inertia	− 10 min	50	55.6 (9.6)	50	55.5 (10.2)
Vigor-activity	− 10 min	50	45.3 (8.9)	50	45.7 (8.6)
Nutritional laboratory test values					
Hemoglobin (g/dL)	Screening	50	13.8 (1.3)	50	13.6 (1.3)
Hematocrit (%)	Screening	50	42.3 (3.3)	50	42.1 (3.5)
Total protein (g/dL)	Screening	50	7.2 (0.4)	50	7.1 (0.4)
Albumin (g/dL)	Screening	50	4.5 (0.3)	50	4.4 (0.3)
VAS					
Positive feelings	− 10 min	50	51.6 (16.8)	49	50.6 (19.2)
Clear thinking	− 10 min	50	55.9 (20.4)	49	54.6 (19.9)
Motivation	− 10 min	50	51.6 (20.7)	49	50.3 (19.3)
Concentration	− 10 min	50	52.7 (20.4)	49	53.7 (22.0)
Persistence	− 10 min	50	51.2 (19.7)	49	48.2 (19.7)
Energy	− 10 min	50	54.8 (21.0)	49	56.1 (20.4)
Progress	− 10 min	50	55.6 (19.6)	49	57.0 (19.5)
Relaxation	− 10 min	50	47.9 (20.8)	49	47.2 (22.0)
Tiredness	− 10 min	50	56.0 (19.9)	49	58.1 (18.6)

### Changes in profile of mood states 2nd edition t-scores

**Fig. 3:**
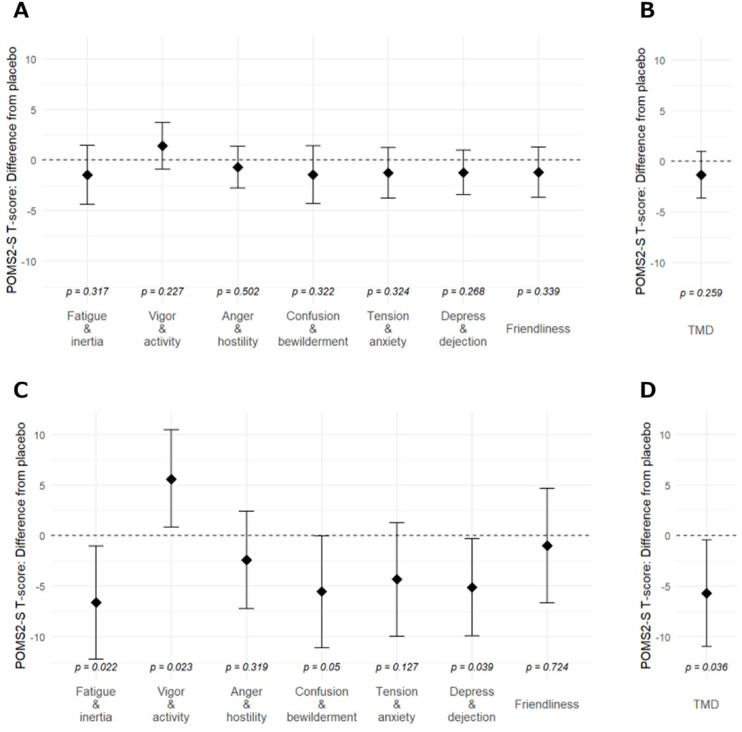
Least squares mean differences in changes from baseline in POMS2-S T-scores for FI, VA, AH, CB, TA, DD, and F with the L-histidine group, compared with the placebo group, in the all participants analysis set (**A**) and subgroup with high fatigue levels: POMS2-S FI T-score ≥ 60 at baseline (**C**). Least squares mean differences in changes from baseline in POMS2-S T-scores (each group n = 50) for TMD with the L-histidine group, compared with the placebo group, in the all participants analysis set (**B**) and subgroup with high fatigue levels: POMS2-S FI T-score ≥ 60 at baseline (**D**). Graphs show point estimates (filled diamonds) with 95% confidence intervals (vertical bar).

The POMS2-S T-scores at baseline and postingestion assessment (90 min after ingestion) are shown in Table 2, and the changes in POMS2-S T-scores from baseline to postingestion assessment (90 min after ingestion) are shown in Fig. 3 and Fig. S1. In the all participants analysis set, no significant between-group differences were observed in the primary indices of FI and VA or in the changes in AH, CB, DD, TA, F, and TMD, which were secondary indices. Similarly, in the change from baseline to 140 min, no significant between-group differences were observed in any of the endpoints in the all participants analysis set (Tables S1 and S2).

**Table 2:** Changes in POMS2-S T-scores in the L-histidine or placebo group (baseline and 90 min).

Mood	Capsule	Baseline		90 min	
		n	Mean (SD)	n	Mean (SD)
All participants					
Fatigue-inertia (FI)	L-histidine	50	55.6 (9.6)	50	53.1 (8.5)
	Placebo	50	55.5 (10.2)	50	54.5 (10.0)
Vigor-activity (VA)	L-histidine	50	45.3 (8.9)	50	46.8 (7.8)
	Placebo	50	45.7 (8.6)	50	45.7 (8.9)
Anger-hostility (AH)	L-histidine	50	49.1 (8.6)	50	43.8 (6.7)
	Placebo	50	48.5 (9.6)	50	44.2 (6.8)
Confusion-bewilderment (CB)	L-histidine	50	56.8 (9.6)	50	51.7 (7.3)
	Placebo	50	53.8 (8.3)	50	51.3 (9.9)
Tension-anxiety (TA)	L-histidine	50	55.1 (9.7)	50	49.7 (7.6)
	Placebo	50	53.4 (9.3)	50	50.0 (8.7)
Depress-dejection (DD)	L-histidine	50	52.5 (9.4)	50	47.8 (6.3)
	Placebo	50	50.8 (8.3)	50	48.1 (8.2)
Friendliness (F)	L-histidine	50	47.6 (9.0)	50	43.5 (9.0)
	Placebo	50	48.1 (9.4)	50	45.1 (10.6)
Total mood disturbance (TMD)	L-histidine	50	55.1 (9.4)	50	50.1 (6.7)
	Placebo	50	53.6 (8.2)	50	50.7 (8.1)
Subgroup: FI T-score ≧60					
Fatigue-inertia(FI)	L-histidine	15	67.6 (6.8)	15	57.0 (9.8)
	Placebo	19	67.1 (4.6)	19	63.3 (7.0)
Vigor-activity (VA)	L-histidine	15	40.9 (6.2)	15	47.5 (8.1)
	Placebo	19	46.9 (8.7)	19	47.1 (9.9)
Anger-hostility (AH)	L-histidine	15	55.3 (10.0)	15	46.7 (9.9)
	Placebo	19	53.3 (11.9)	19	48.2 (7.6)
Confusion-bewilderment (CB)	L-histidine	15	64.5 (9.7)	15	54.8 (8.6)
	Placebo	19	59.6 (8.5)	19	57.1 (10.3)
Tension-anxiety (TA)	L-histidine	15	62.9 (9.4)	15	52.9 (10.4)
	Placebo	19	60.8 (7.8)	19	56.0 (8.5)
Depress-dejection (DD)	L-histidine	15	60.2 (10.3)	15	51.6 (7.8)
	Placebo	19	54.1 (10.6)	19	52.7 (10.4)
Friendliness (F)	L-histidine	15	44.1 (8.7)	15	41.5 (10.8)
	Placebo	19	49.4 (9.3)	19	46.9 (10.8)
Total mood disturbance (TMD)	L-histidine	15	64.9 (8.7)	15	53.5 (9.3)
	Placebo	19	60.4 (8.5)	19	56.5 (8.2)

In the subgroup with high fatigue levels (POMS2-S FI T-score ≥ 60 at baseline), the L-histidine group showed significant decreases compared with the placebo group in the changes in FI, CB, DD, and TMD, and the L-histidine group showed a significant increase compared with the placebo group in the change in VA.

### Changes in visual analog scale scores

**Fig. 4:**
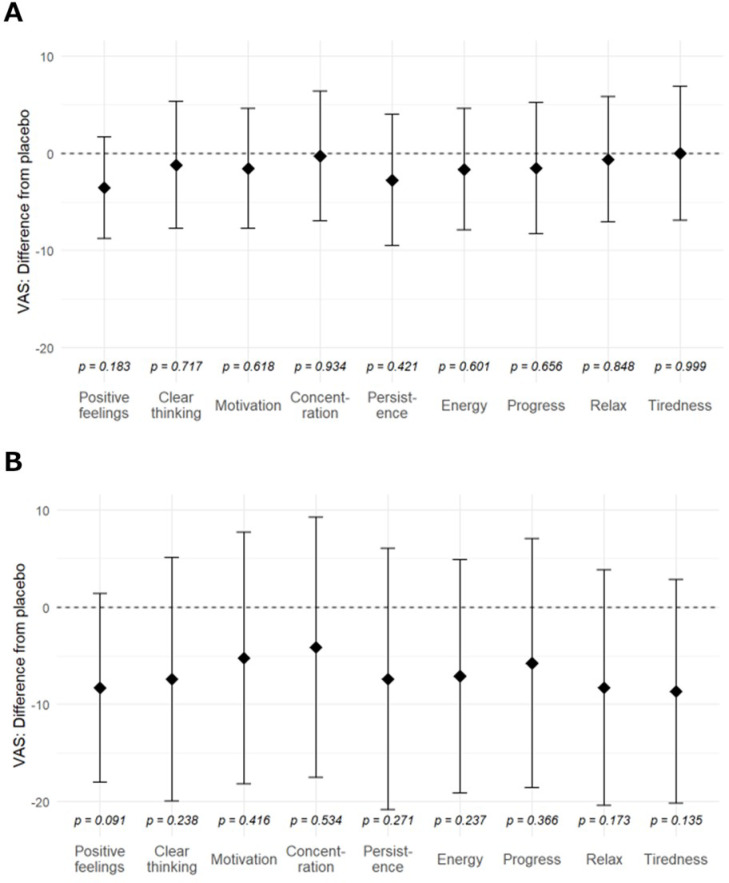
Least squares mean differences in changes from baseline in VAS scores for positive feelings, clear thinking, motivation, concentration, persistence, energy, progress, relaxation, and tiredness with the L-histidine group, compared with the placebo group, in the overall participants analysis set (**A**) and subgroup with high fatigue levels: POMS2-S FI T-score ≥ 60 at baseline (**B**). Graphs show point estimates (filled diamonds) with 95% confidence intervals (vertical bar). A negative change indicates an improvement in mood state in the L-histidine group compared to the placebo group (as mean values).

The VAS scores at baseline and postingestion assessment (90 min after ingestion) are shown in Table 3, and the change in the VAS score from baseline to postingestion assessment (90 min after ingestion) is shown in Fig. 4 and Fig. S2. No significant between-group differences were observed in any of the nine VAS scores on the all participants analysis set. Similarly, in the changes from baseline to 30 and 140 min, no significant between-group differences were observed in any of the endpoints of the all participants analysis set (Tables S3 and S4).

**Table 3:** Changes in nine temporary moods evaluated with VAS scores in the L-histidine or placebo group (baseline and 90 min).

Question items	Capsule	Baseline		90 min	
		n	Mean (SD)	n	Mean (SD)
All participants					
Positive feelings	L-histidine	50	51.6 (16.8)	49	46.1 (14.3)
	Placebo	49	50.6 (19.2)	50	49.1 (19.2)
Clear thinking	L-histidine	50	55.9 (20.4)	49	47.6 (18.7)
	Placebo	49	54.6 (19.9)	50	48.3 (19.6)
Motivation	L-histidine	50	51.6 (20.7)	49	46.8 (19.1)
	Placebo	49	50.3 (19.3)	50	47.8 (20.5)
Concentration	L-histidine	50	52.7 (20.4)	49	46.7 (20.0)
	Placebo	49	53.7 (22.0)	50	47.7 (20.6)
Persistence	L-histidine	50	51.2 (19.7)	49	46.1 (20.3)
	Placebo	49	48.2 (19.7)	50	47.3 (21.4)
Energy	L-histidine	50	54.8 (21.0)	49	49.6 (18.3)
	Placebo	49	56.1 (20.4)	50	52.1 (19.4)
Progress	L-histidine	50	55.6 (19.6)	49	48.5 (19.2)
	Placebo	49	57.0 (19.5)	50	50.9 (18.8)
Relaxation	L-histidine	50	47.9 (20.8)	49	45.6 (21.3)
	Placebo	49	47.2 (22.0)	50	45.3 (22.6)
Tiredness	L-histidine	50	56.0 (19.9)	49	58.6 (17.2)
	Placebo	49	58.1 (18.6)	50	59.4 (19.4)
Subgroup: FI T-score ≧60					
Positive feelings	L-histidine	15	61.3 (15.8)	15	51.4 (12.7)
	Placebo	19	57.3 (19.2)	19	57.0 (21.1)
Clear thinking	L-histidine	15	67.2 (17.1)	15	50.8 (14.5)
	Placebo	19	63.3 (19.3)	19	56.7 (21.6)
Motivation	L-histidine	15	63.6 (18.9)	15	51.1 (15.6)
	Placebo	19	60.6 (17.0)	19	55.0 (22.4)
Concentration	L-histidine	15	62.2 (18.5)	15	49.4 (15.9)
	Placebo	19	65.6 (20.3)	19	54.8 (22.6)
Persistence	L-histidine	15	61.3 (17.3)	15	49.2 (18.3)
	Placebo	19	55.4 (20.9)	19	53.2 (24.0)
Energy	L-histidine	15	63.6 (19.2)	15	50.2 (15.8)
	Placebo	19	64.1 (18.0)	19	57.6 (21.0)
Progress	L-histidine	15	66.1 (16.8)	15	50.7 (15.3)
	Placebo	19	66.2 (17.1)	19	56.5 (21.0)
Relaxation	L-histidine	15	61.5 (19.1)	15	47.2 (19.1)
	Placebo	19	54.2 (22.5)	19	50.5 (24.2)
Tiredness	L-histidine	15	70.0 (15.1)	15	57.8 (16.7)
	Placebo	19	69.3 (14.8)	19	66.3 (17.3)

Even in the subgroup with high fatigue levels (POMS2-S FI T-score ≥ 60 at baseline), no significant between-group differences were observed in the change in any of the nine VAS.

### Cognitive assessment

The results of the 12 indices calculated using Cognitrax at baseline (first time) and postingestion assessment (second time) are presented in Table S5. The changes in these 12 indices from the baseline (first time) to postingestion assessment (second time) are shown in Fig. S3 and Fig. S4. In the all participants analysis set, the placebo group showed significant increases compared with the L-histidine group in changes in psychomotor speed and processing speed (psychomotor speed: L-histidine group 3.1 [11.4], placebo group 5.8 [8.8], *p* = 0.039; processing speed: L-histidine group 1.4 [15.8], placebo group 6.5 [12.8], *p* = 0.020). No statistically significant between-group differences were observed for any of the other indices.

In the subgroup with high fatigue levels (POMS2-S FI T-score ≥ 60 at baseline), no significant between-group differences were observed in changes in any of the 12 indices.

### Uchida–Kraepelin performance test

The number of responses, number of correct responses, and percentage of correct responses in the first and second halves of the UK-test are presented in Table S6. No significant differences were observed between groups in either the all participants analysis set or subgroup with high fatigue levels (POMS2-S FI T-score ≥ 60 at baseline).

### Safety assessment

No adverse events and side effects were observed in any of the participants.

## Discussion

In the current study, we evaluated the effect of a single dose of L-histidine on improving mood states, such as mental fatigue and lack of vigor caused by simple calculation tasks. A prespecified analysis of the all participants analysis set revealed no significant improvement in the L-histidine group compared with the placebo group for the primary endpoint (primary indices FI and VA, and secondary indices other POMS-2 T-scores) nor the secondary endpoints (VAS, Cognitrax, and UK-test scores). However, although these were exploratory results, the subgroup analysis in the “high fatigue levels” (POMS2-S FI T-score ≥ 60 at baseline) showed significant decreases in the L-histidine group for the primary endpoint, POMS2-S T-scores, changes in FI, CB, DD, and TMD, and a significant increase in VA.

Because a previous human study that showed a positive effect of 1.65 g L-histidine ingestion for 2 weeks on mental fatigue^17^ targeted participants with high fatigue levels, this study protocol prioritized the inclusion of “participants with high fatigue levels.” However, participant recruitment did not result in the inclusion of only those with high fatigue levels. Therefore, analyses were performed on the subgroup limited to the high-fatigue group. The POMS2-S manual defines FI T-score as “ < 30: very low, 30–39: low, 40–59: average, 60–69: high, and 70 ≤ : very high.” Based on this stratification, the subgroup with high fatigue levels in this study was defined as those with POMS2-S FI T-score ≥ 60 at baseline. A previous study^17^ used the POMS (full version), a pre-revision of the POMS2 questionnaire, as the primary endpoint. The POMS (full version) and POMS2-S differed in one of the five fatigue category questions. In a previous study, the inclusion criterion for participant selection was a raw score of ≥ 16 (when converted to T-scores, it is equivalent to “ ≥ 60.”) on the POMS (full version). Therefore, in terms of the degree of fatigue, the subgroup with high fatigue levels in the current study was almost the same as that of participants included in the previous study. In this study, we recruited "healthy men and women who regularly experienced mental fatigue and sleep deprivation" as participants. However, their baseline POMS2-S T-Scores were within the average range (L-histidine group: 55.6 (9.6), Placebo group: 55.5 (10.2); see Table 1). The following two factors may be responsible for this. The first factor is the difference in the assessment method. Participants were asked whether they met the inclusion criterion "healthy men and women who regularly experience mental fatigue and sleep deprivation." Meanwhile, in the POMS2-S FI, participants’ fatigue levels were assessed multifacetedly based on multiple questions. The second factor is the timing of the assessment. While participants were asked about their “daily” fatigue state based on the inclusion criterion, they also answered using the POMS2-S based on their state “at the time of the test”. Thus, participants in this study included those who met the inclusion criteria but did not experience high fatigue levels specified by POMS2-S FI assessment at the time of the test.

As described in Introduction, histamine produced in the brain by L-histidine ingestion is involved in various brain functions and acts as a neurotransmitter via four types of histamine receptors (H1R, H2R, H3R, and H4R) in the brain^8^. H1R is present throughout the central nervous system and promotes wakefulness^7,8,10–12^. Therefore, it is reasonable that L-histidine ingestion increases brain histamine, which induces wakefulness via H1R, thereby improving fatigue and vigor caused by computational workload. Animal studies have shown that in a state of high fatigue under conditions of sleep deprivation due to water-floor stress, histamine levels in the brain are reduced, and oral administration of L-histidine improves memory impairment by increasing histamine levels in the brain^16^. Therefore, as observed in this study, it is scientifically reasonable that the beneficial effects of L-histidine on “fatigue, vitality/energy, confusion/bewilderment (including mental clarity), depression/depression, and overall negative mood states” are more likely to be observed in participants with high fatigue levels.

The L-histidine group also showed a significant decrease in the change in the POMS2-S CB T-score, the secondary index of the primary endpoint, compared with the placebo group in the subgroup with high fatigue levels. The CB T-score is a standardized assessment of five items converted into a T-score, and the POMS2 manual indicates that a higher CB T-score indicates “confusion and inability to organize one’s thoughts.” Furthermore, the POMS2 Assessment Report describes a high confusion score as “moments of confusion or unclear thinking,” suggesting that a decrease in the POMS2-S CB T-score indicates an improvement to “clear thinking.” In a 2-week L-histidine ingestion study^17^, the L-histidine group showed significant improvement compared with the placebo group in “clear thinking” evaluated using the VAS. In this study, although no significant difference was observed between the groups even in the subgroup with high fatigue levels, the mean score showed improvement in the L-histidine group (L-histidine group, –16.4 [20.9]; placebo group, –6.58 [20.7], *p* = 0.238, with a lower score indicating a state of stronger “clear thinking”). Although more comprehensive assessments are required, in individuals with high fatigue levels, the single dose of L-histidine might be effective in improving “clear thinking” as well as 2 weeks of continuous ingestion.

Furthermore, the L-histidine group showed significant improvements compared with the placebo group in the primary endpoint of the POMS2-S, including changes in VA, DD, and TMD in the subgroup with high fatigue levels. Therefore, it is possible that the single dose of L-histidine may be effective in improving mood states, such as “I feel energized and positive,” and mood states, such as “I feel worthless, unable to cope, lonely, sad, and guilty,” and “I currently have problems with emotional functioning.” A randomized, double-blind, placebo-controlled crossover study in women who ingested DBB, a compound rich in L-histidine, for 2 weeks also found that DBB significantly reduced negative mood factors, such as AH, confusion, DD, fatigue, TA, and TMD, compared with the placebo group, as assessed using the POMS^22^. In addition, DBB significantly increased the positive mood factor vigor compared with placebo. In another placebo-controlled, double-blind, crossover study conducted on 56 healthy adults with high fatigue scores on the POMS test, who were 30–60 years old, 35 men (Fatigue raw score ≥ 16) and 21 women (Fatigue raw score ≥ 17), 4 weeks of DBB ingestion was reported^20^. Although the results showed no significant differences between groups, the DBB group had significantly lower POMS fatigue and TMD scores after 4 weeks compared with baseline, whereas the placebo group showed no significant changes. Furthermore, a meta-analysis^32^ based on 18 studies involving 776 participants reported that histidine-containing dipeptide ingestion (anserine/carnosine, L-carnosine, and β-alanine) significantly reduced depression scores (Beck Depression Inventory) and increased quality of life scores (SF-36) compared with placebo ingestion. These results are consistent with those of the current study, suggesting that L-histidine ingestion during high fatigue may have a positive effect on various mood states. This suggestion is considered reasonable from the perspective of the mechanism of action, in which L-histidine is synthesized into histamine in cells of the posterior hypothalamus, which have projections to various brain regions^33^ and regulates various functions, such as the sleep–wake cycle, appetite, memory, and stress response^34^.

In the subgroup with high fatigue levels, although no significant improvement by L-histidine ingestion was observed on the VAS, mean scores for all items decreased (in the direction of improvement) in the L-histidine group compared to the placebo group (Fig. 4B). Therefore, both the POMS2-S and VAS appear to show a consistent trend toward improvement in subjective mood. Mood states (POMS2-S and VAS) are subjective and therefore susceptible to potential expectancy or placebo effects. However, this study was a double-blind comparative study, and the test foods were capsules that were indistinguishable in appearance. Furthermore, in both the subjective POMS2-S and VAS, the L-histidine group showed greater improvement than the placebo group in all assessment items in the subgroup with high fatigue levels (as mean changes from baseline) (Figs. 3B, 4B). Cognitive performance assessment using Cognitrax revealed no significant differences between the groups in the NCI, a comprehensive score of cognitive function, in either the all participants analysis set or the subgroup with high fatigue levels. H2R agonists enhance synaptic transmission in the hippocampus and increase the firing rate of many types of neurons^11^. In addition, central histaminergic neurons project to the PFC, a central area for executive function^16^. Given these findings, memory and executive function were considered potential targets for L-histidine; however, this study did not demonstrate any effect of L-histidine on them. In a study in which mice were administered DBB 1.6 g/kg body weight or L-histidine (500 mg/kg body weight), the animals’ exploratory behavior toward novel objects significantly increased, and the spontaneous alternation rate in a Y-maze under scopolamine-induced amnesia conditions significantly increased, suggesting that L-histidine improves short-term spatial memory^35^. In addition, a systematic review and meta-analysis of randomized controlled trials investigating the effects of histidine-containing dipeptide (a combination of anserine and carnosine) ingestion on human cognitive performance demonstrated that the administration of 500–1000 mg of histidine-containing dipeptides daily for at least 3 months improved delayed recall on the Wechsler Memory Scale in both patients with MCI and cognitively normal participants^36^. Furthermore, a previous study on 2-week L-histidine daily ingestion showed significant improvements in “visual learning,” one of the measures obtained from the CogHealth cognitive function test, compared with the placebo group^17^. As the related assessment, “Visual Memory,” one of the measures obtained from the Cognitrax, was used as an evaluation measure in the current study. Although the two evaluations are similar, visual learning in CogHealth evaluates immediate recall ability, whereas visual memory in Cognitrax also considers delayed recall ability, which means that these indices evaluate different functions. Therefore, the results of the single dose in this study do not contradict those of the previous study. Since the primary endpoints of this study were POMS-2-S FI (Fatigue-inertia) and VA (Vigor-activity), priority was given to including participants with high levels of fatigue, and those with impaired cognitive function were not included as inclusion criteria. To clarify the effects of the single dose of L-histidine on cognitive function, further studies that consider appropriate participant selection and intervention periods are required.No adverse events were observed in either the L-histidine or placebo group in this study. Clinical studies on L-histidine in patients with anemia due to uremia or chronic dialysis^37^ (8 weeks, 1 g/day, 6 participants), healthy adult men^38^ (2 weeks, 4 g/day, 8 participants), patients with rheumatoid arthritis^39^ (single dose, 3.7 g per dose, 26 participants), and healthy men experiencing daily fatigue and sleep deprivation^19^ (2 weeks, 1.65 g/day, 20 participants) revealed no significant safety issues. Furthermore, no significant health problems were reported with a commercially available product of the same dose of L-histidine (1.65 g per dose) used in this study, “Maiasa Histidine®” (dosage: once daily, taken daily), which has been sold in Japan since 2017 as a food with functional claims (data not shown).

This study has some limitations. Firstly, the superiority of L-histidine over placebo was confirmed in the subgroup with high fatigue levels (POMS2-S FI T-score ≥ 60 at baseline) in terms of mood states, such as mental fatigue and vigor; however, the number of participants in this subgroup was 34, which was not a sample size designed for this purpose. Furthermore, no objective or biological markers were measured to support the changes in mood states observed through subjective assessment. Therefore, to verify the reliability of the results in participants with high fatigue levels, further validation studies with larger sample sizes are necessary. Therefore, it should be noted that these results are the result of an exploratory consideration. Secondly, this study was conducted as a randomized, double-blind, placebo-controlled trial designed to minimize placebo effects. However, POMS2-S and VAS are self-reported, subjective assessments, and participants’ expectations of improvement from the intervention may have influenced not only the L-histidine group but also the placebo group. It is possible that some participants were affected by these expectancy and/or placebo effects, which increased variability and might have reduced statistical power. Therefore, future validation studies need to adopt a study design that minimizes to the greatest extent possible these effects that may have occurred in both groups. Thirdly, although this study was designed to induce a fatigue load state using the UK-test, the degree of fatigue load may have varied among participants owing to participant characteristics, including calculation ability. Further investigation is necessary after inducing a uniform fatigue load. Finally, since this study did not include a familiarization session or counterbalancing for the cognitive task, the results of Cognitrax, a secondary endpoint, require caution in interpreting the results. In the all-participants analysis set, the placebo group showed significant increases in changes in psychomotor speed and processing speed compared to the L-histidine group. However, this may reflect the practice effects rather than the effects of L-histidine, and further investigation is needed to verify the L-histidine effect on cognitive function.

Although this study did not demonstrate the significant positive effects of the L-histidine group over the placebo group in all endpoints in the prespecified all participants analysis set, it suggests for the first time that the single dose of L-histidine may be effective in alleviating the negative mood states, such as FI, CB (including unclear thinking), DD, and overall TMD, and in improving VA after a workload in participants with high fatigue levels (POMS2-S FI T-score ≥ 60 at baseline). Although further validation is required, these results are consistent with those of a previous study (2-week continuous L-histidine ingestion)^17^, animal studies, and the mechanism of action. No adverse events or safety issues were observed. Caffeine and sugar are commonly used to reduce fatigue and drowsiness, and energy drinks are particularly popular among children, adolescents, and young adults. However, there are health concerns regarding the daily consumption of these substances in large amounts^40–42^. The use of alternatives with different mechanisms of action, such as L-histidine, is of social importance. Future investigations are expected to explore the effects of L-histidine as a substitute for caffeine and sugar and their synergistic effects in combination with them.

## Conclusion

Although a single ingestion of L-histidine showed no significant improvements in any of the endpoints including the primary indices FI and VA compared with the placebo in the prespecified all-participants analysis set, the exploratory findings suggest that it may alleviate negative mood states, including fatigue, confusion (including unclear thinking), and depression, and may also improve vigor after a workload in humans with high fatigue levels.

## Supplementary Information

Below is the link to the electronic supplementary material.

Supplementary Material 1.

Supplementary Material 2.

Supplementary Material 3.

Supplementary Material 4.

Supplementary Material 5.

## Data Availability

Data from the study in a summarized format, trial protocol and statistical analysis plan can be made available upon reasonable request.

## Abbreviations

FI: Fatigue-inertia
CB: Confusion-bewilderment
DD: Depression-dejection
TMD: Total mood disturbance
VA: Vigor-activity
PFC: Prefrontal cortex
DBB: Dried bonito broth
POMS2-S: Profile of Mood States 2nd Edition-Short
AIS-J: Japanese version of the Athens Insomnia Scale
VAS: Visual analog scale
AH: Anger-hostility
TA: Tension-anxiety
F: Friendliness
FAS: Full analysis set
SAS: Safety analysis set
NCI: Neurocognitive index
