# Supplementary material for: Effects of a single dose of L-histidine on mental fatigue and vigor in participants with high fatigue levels: a randomized controlled trial
Source: Sci Rep. 2026 Apr 15;16:17553. doi: 10.1038/s41598-026-48060-x (PMC13243660; doi:10.1038/s41598-026-48060-x)
Supplement: Supplementary file 1 — Supplementary Material 1. [file 41598_2026_48060_MOESM1_ESM.docx]

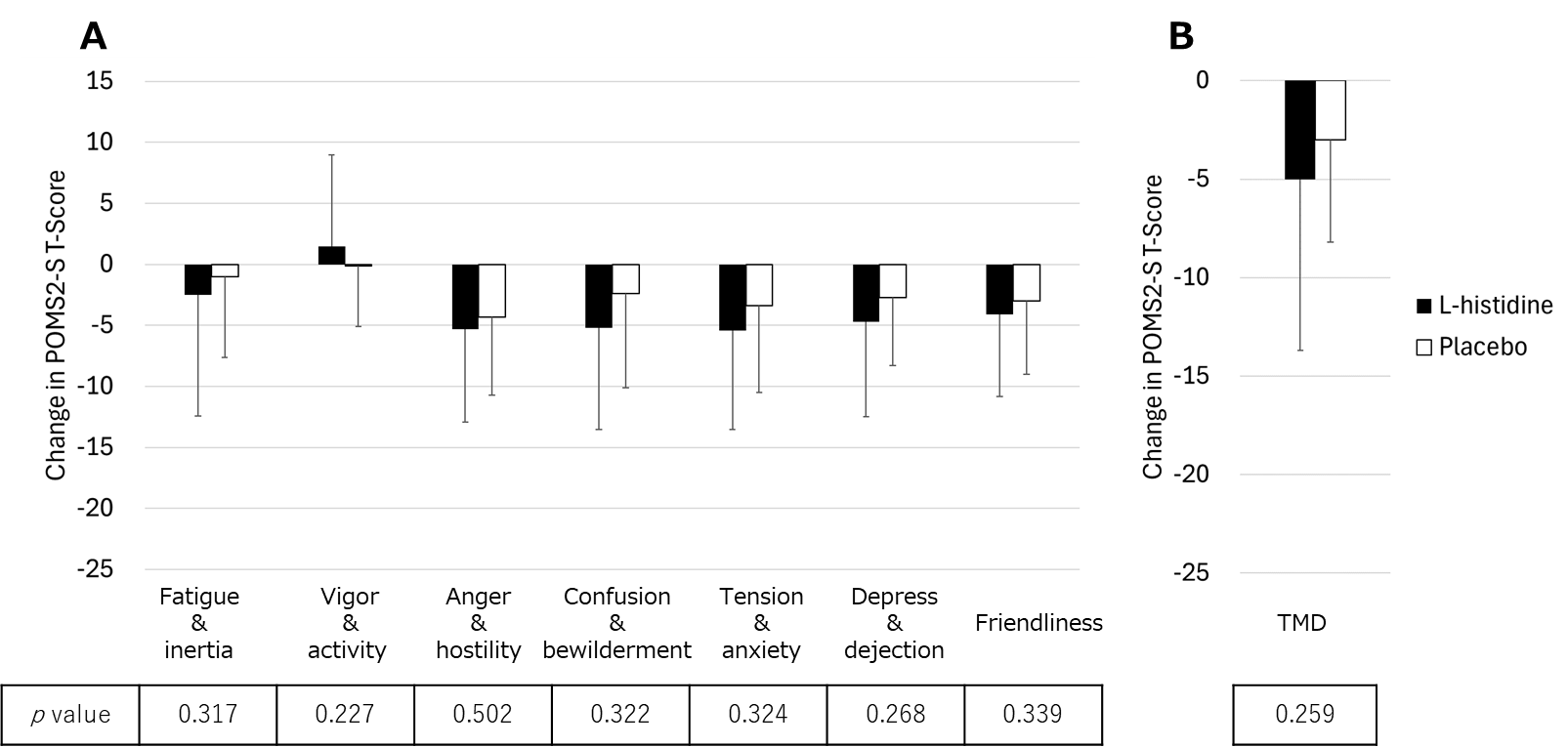


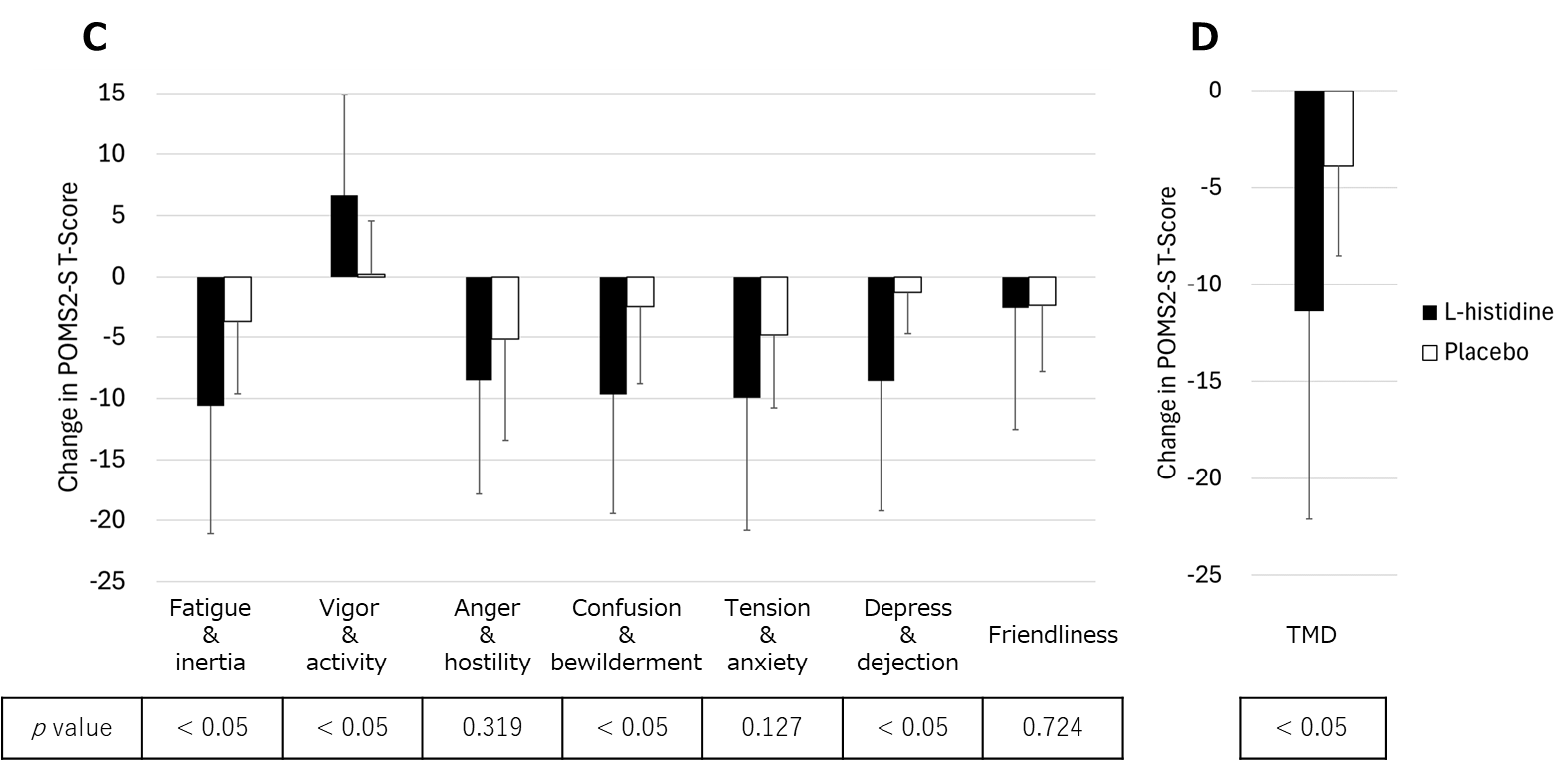


**Fig. S1.** Changes in POMS2-S T-scores from baseline following L-histidine (black) or placebo (white) ingestion for FI, VA, AH, CB, TA, DD, and F in the all participants analysis set (A) and subgroup with high fatigue levels: POMS2-S FI T-score ≥ 60 at baseline (C). Changes in POMS2-S T-scores from baseline following L-histidine (black) or placebo (white) ingestion (each group n = 50) for TMD in the all participants analysis set (B) and subgroup with high fatigue levels: POMS2-S FI T-score ≥ 60 at baseline (D). Values are expressed as means (SDs).
