# Supplementary material for: Effects of a single dose of L-histidine on mental fatigue and vigor in participants with high fatigue levels: a randomized controlled trial
Source: Sci Rep. 2026 Apr 15;16:17553. doi: 10.1038/s41598-026-48060-x (PMC13243660; doi:10.1038/s41598-026-48060-x)
Supplement: Supplementary file 2 — Supplementary Material 2. [file 41598_2026_48060_MOESM2_ESM.docx]

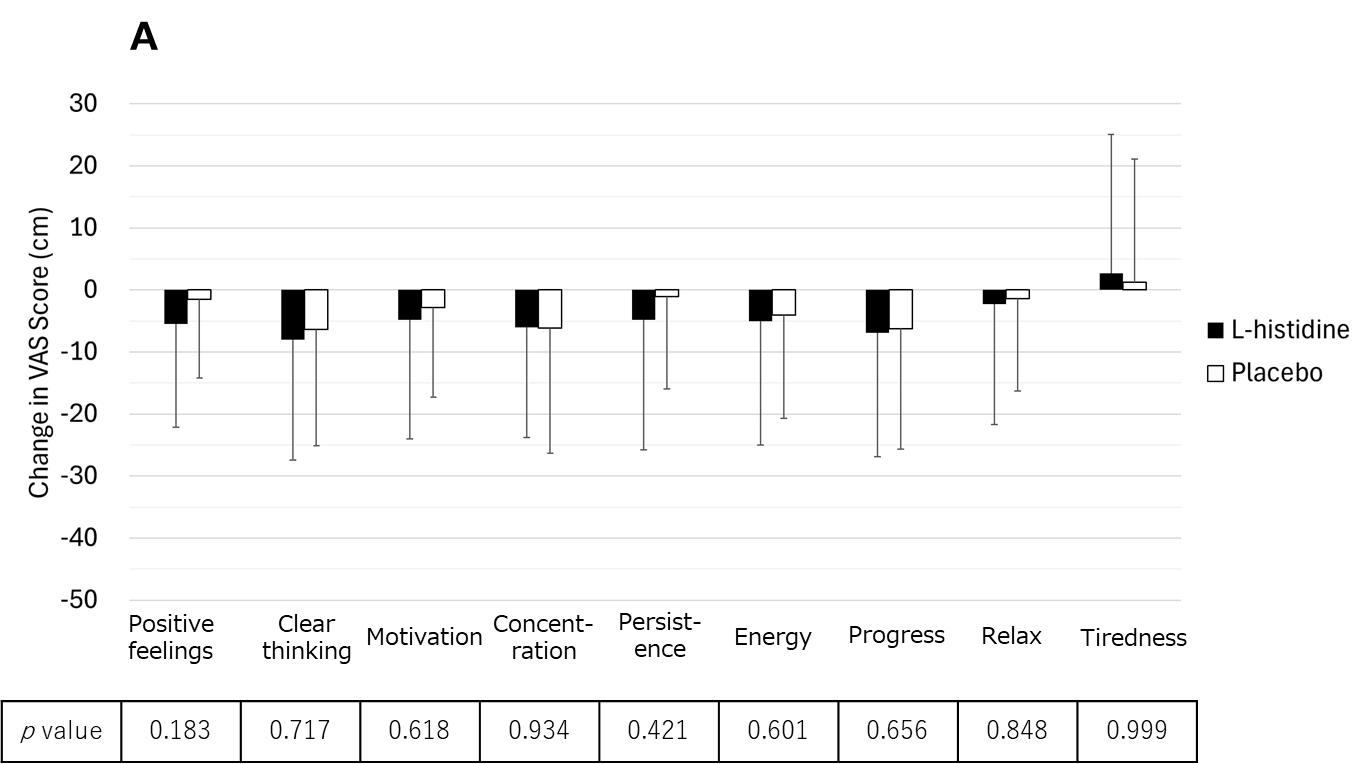


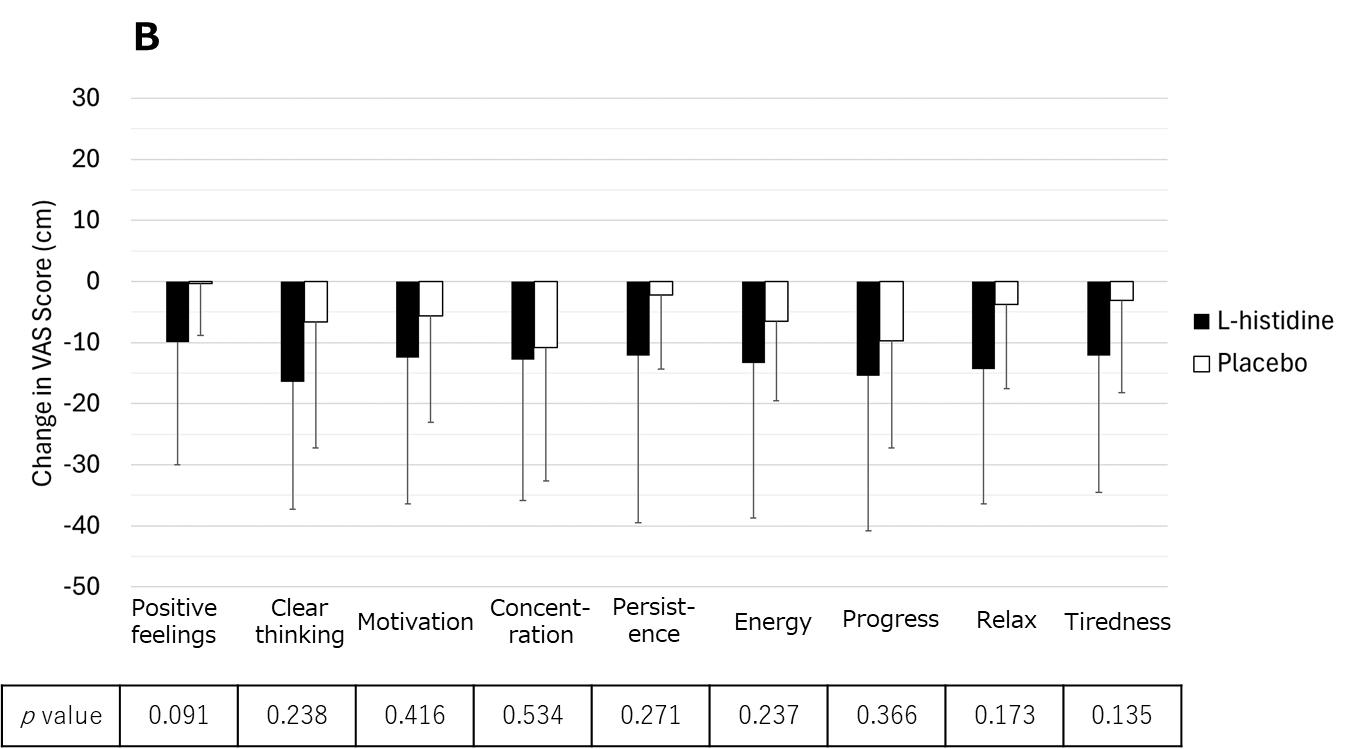


**Fig. S2.** Changes in VAS scores from baseline following L-histidine (black) or placebo (white) ingestion for positive feelings, clear thinking, motivation, concentration, persistence, energy, progress, relaxation, and tiredness in the overall participants analysis set (A) and subgroup with high fatigue levels: POMS2-S FI T-score ≥ 60 at baseline (B). Values are expressed as means (SDs). A negative change indicates an improvement in mood state.
