# Supplementary material for: Effects of a single dose of L-histidine on mental fatigue and vigor in participants with high fatigue levels: a randomized controlled trial
Source: Sci Rep. 2026 Apr 15;16:17553. doi: 10.1038/s41598-026-48060-x (PMC13243660; doi:10.1038/s41598-026-48060-x)
Supplement: Supplementary file 3 — Supplementary Material 3. [file 41598_2026_48060_MOESM3_ESM.docx]

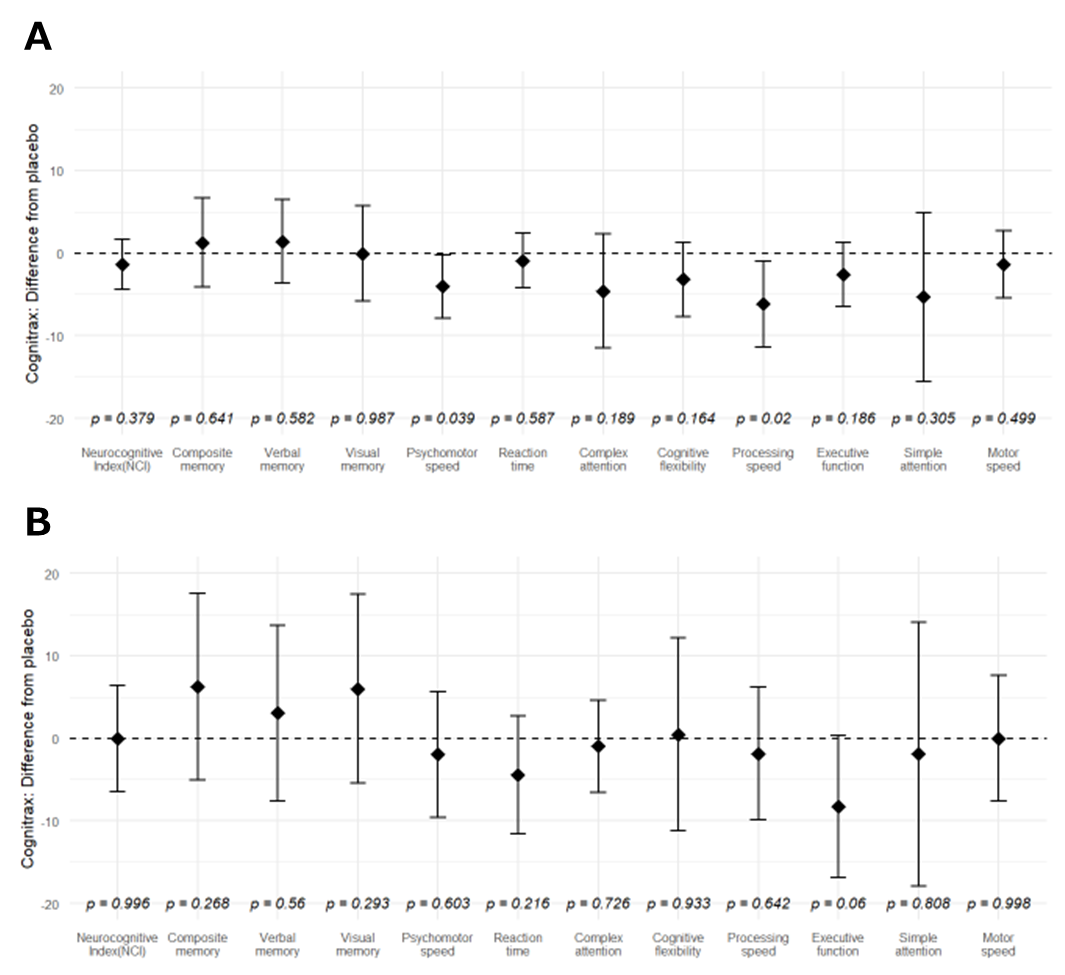


**Fig. S3.** Least squares mean differences in changes from baseline in the Cognitrax for NCI, composite memory, verbal memory, visual memory, psychomotor speed, reaction time, complex attention, cognitive flexibility, processing speed, executive function, simple attention, and motor speed with the L-histidine group, compared with the placebo group, in the all participants analysis set (A) and subgroup with high fatigue levels: POMS2-S FI T-score ≥ 60 at baseline (B). Graphs show point estimates (filled diamonds) with 95% confidence intervals (vertical bar).
