# Supplementary material for: Effects of a single dose of L-histidine on mental fatigue and vigor in participants with high fatigue levels: a randomized controlled trial
Source: Sci Rep. 2026 Apr 15;16:17553. doi: 10.1038/s41598-026-48060-x (PMC13243660; doi:10.1038/s41598-026-48060-x)
Supplement: Supplementary file 4 — Supplementary Material 4. [file 41598_2026_48060_MOESM4_ESM.docx]

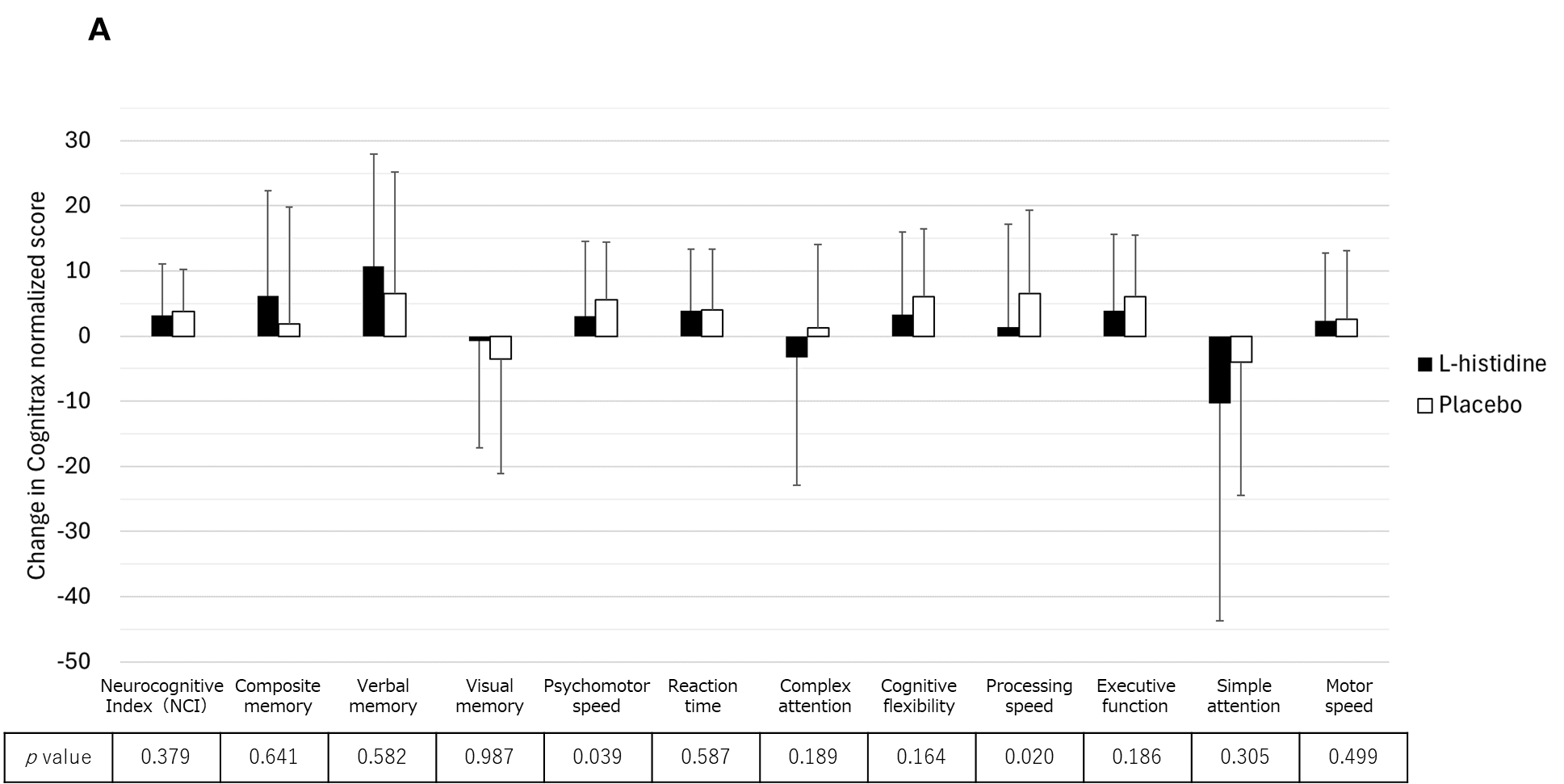


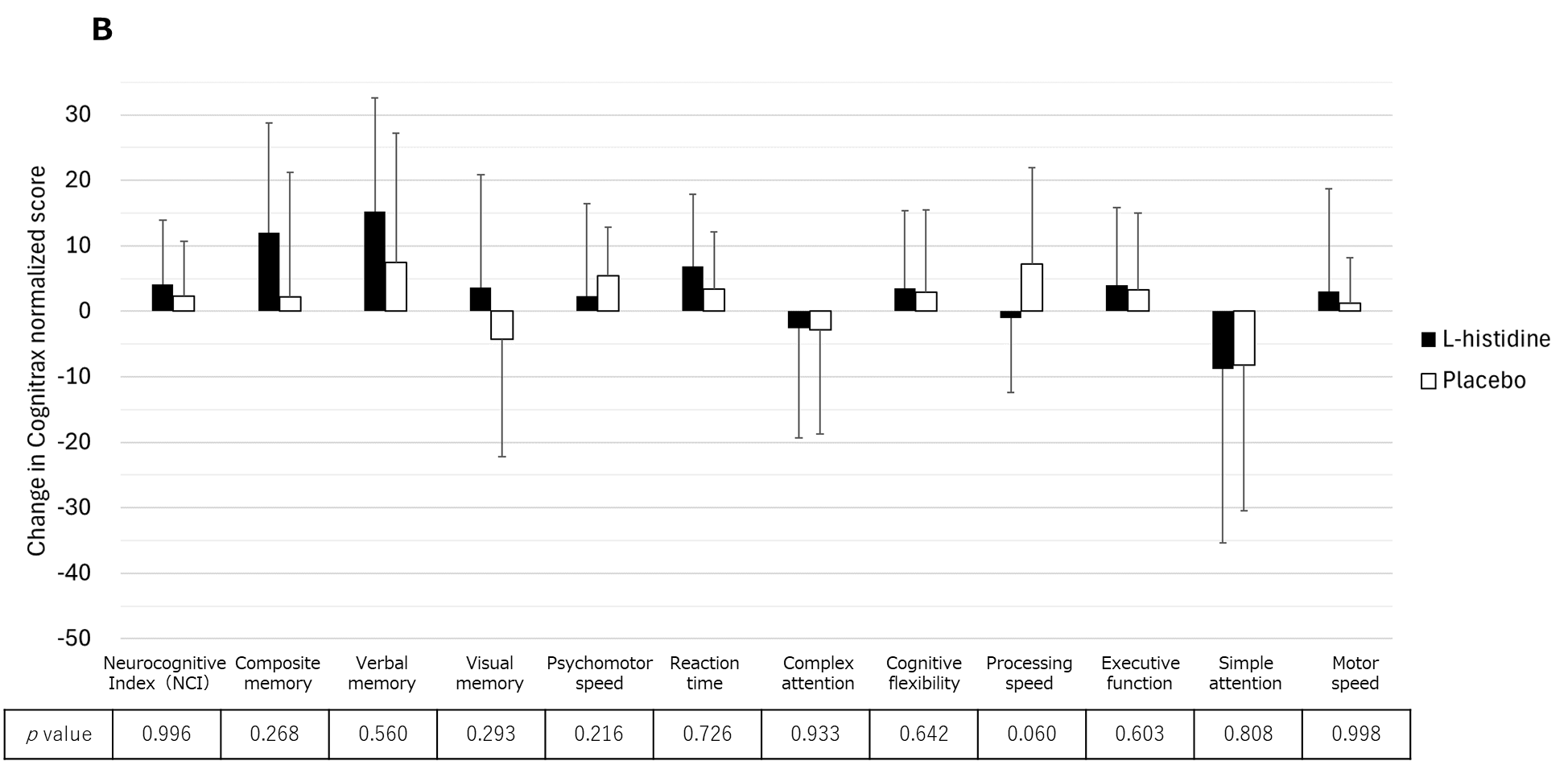


**Fig. S4.** Changes in the Cognitrax subtest results from baseline following L-histidine (black) or placebo (white) ingestion for NCI, composite memory, verbal memory, visual memory, psychomotor speed, reaction time, complex attention, cognitive flexibility, processing speed, executive function, simple attention, and motor speed in the all participants analysis set (A) and subgroup with high fatigue levels: POMS2-S FI T-score ≥ 60 at baseline (B). Values are expressed as means (SDs). A positive change indicates an improvement in cognitive performance.
